# Cigarette smoking induces small airway epithelial epigenetic changes with corresponding modulation of gene expression

**DOI:** 10.1186/1756-8935-6-S1-P10

**Published:** 2013-03-18

**Authors:** Lauren J Buro-Auriemma, Neil R Hackett, Jacqueline Salit, Matthew S Walters, Yael Strulovici-Barel, Michelle R Staudt, Jennifer Fuller, Melisa WY Ho, Ronald G Crystal

**Affiliations:** 1 Department of Genetic Medicine, Weill Cornell Medical College, New York, New York 10065 USA; 2 Inflammation Discovery, Hoffman-La Roche, Nutley, New Jersey 07110 USA

## Background

The small airway epithelium (SAE), the first site of smoking-induced lung pathology, exhibits genome-wide changes in gene expression in response to cigarette smoking. Based on evidence that the epigenome can respond to external stimuli in a rapid manner, we assessed the SAE of smokers for genome-wide DNA methylation changes compared to nonsmokers, and whether these epigenetic changes were linked to the transcriptional program of these cells.

## Materials and methods

SAE was recovered by fiberoptic bronchoscopy and brushing of healthy nonsmokers (n=19) and healthy smokers (n=20). SAE DNA was assessed for genome-wide methylation using the microarray-based high resolution Hpall tiny fragment enriched by ligation-mediated PCR (HELP) assay (Roche-NimbleGen). SAE transcriptome was assessed with Affymetirx HG-U133 Plus 2.0 arrays, with MAS5 and Ingenuity pathway analysis.

## Results

Smoking caused methylation changes of 0.2% of genes distributed across the genome, with the majority characterized by hypomethylation and the minority by hypermethylation. Across the genome, 204 unique genes were differentially methylated in SAE DNA of smokers compared to nonsmokers, with 67% of the differential methylation within 2 kb of transcriptional start sites. For those genes affected, the methylation changes were limited to a focal region of the gene. Among the genes with differential methylation were those related to metabolism, transcription, signal transduction and transport. Smoking induced hypomethylated genes were often related to xenobiotic processes and signal transduction, whereas smoking induced hypermethylated genes represented a wide variety of functions. Among the genes found to be differentially methylated by smoking were genes involved in SAE function of basal stem/progenitor cells, cilia and secretory cells. Of the 204 differentially methylated genes, 34 (17%) correlated with gene expression. Of these, 53% had hypermethylation linked to down-regulation and 47% hypomethylation linked to up-regulation. We also observed the opposite, with hypermethylation associated with up-regulation and hypomethylation linked to down-regulation.

## Conclusions

The data demonstrates that the chronic stress to the lung by cigarette smoking is associated with significant changes in the DNA methylation status of the SAE, a cell population representing <1% of the total lung parenchymal cell population, yet critical to normal lung function and defense. Many of these methylation changes are also linked to alterations of the SAE transcriptional program, indicating that epigenetic modifications likely play an important role in the pathogenesis of smoking-induced lung disease.

